# Supplementary material for: SATB2 drives glioblastoma growth by recruiting CBP to promote FOXM1 expression in glioma stem cells
Source: EMBO Mol Med. 2020 Oct 30;12(12):e12291. doi: 10.15252/emmm.202012291 (PMC7721366; doi:10.15252/emmm.202012291)
Supplement: Supplementary file 1 — Appendix [file EMMM-12-e12291-s001.pdf]

SATB2 Drives Glioblastoma Growth by Recruiting CBP to Promote FOXM1 Expression in Glioma Stem Cells

Weiwei Tao, Aili Zhang, Kui Zhai, Zhi Huang, Haidong Huang, Wenchao Zhou, Qian Huang, Xiaoguang Fang, Briana C. Prager, Xiuxing Wang, Qiulian Wu, Andrew E. Sloan, Manmeet S. Ahluwalia, Justin D. Lathia, Jennifer S. Yu, Jeremy N. Rich, and Shideng Bao

Appendix

Appendix Figure S1

Appendix Figure S2

Appendix Figure S3

Appendix Figure S4

Appendix Figure S5

Appendix Figure S6

Appendix Figure S7

Appendix Figure S8

Appendix Table S1

Appendix Table S2

Appendix Table S3

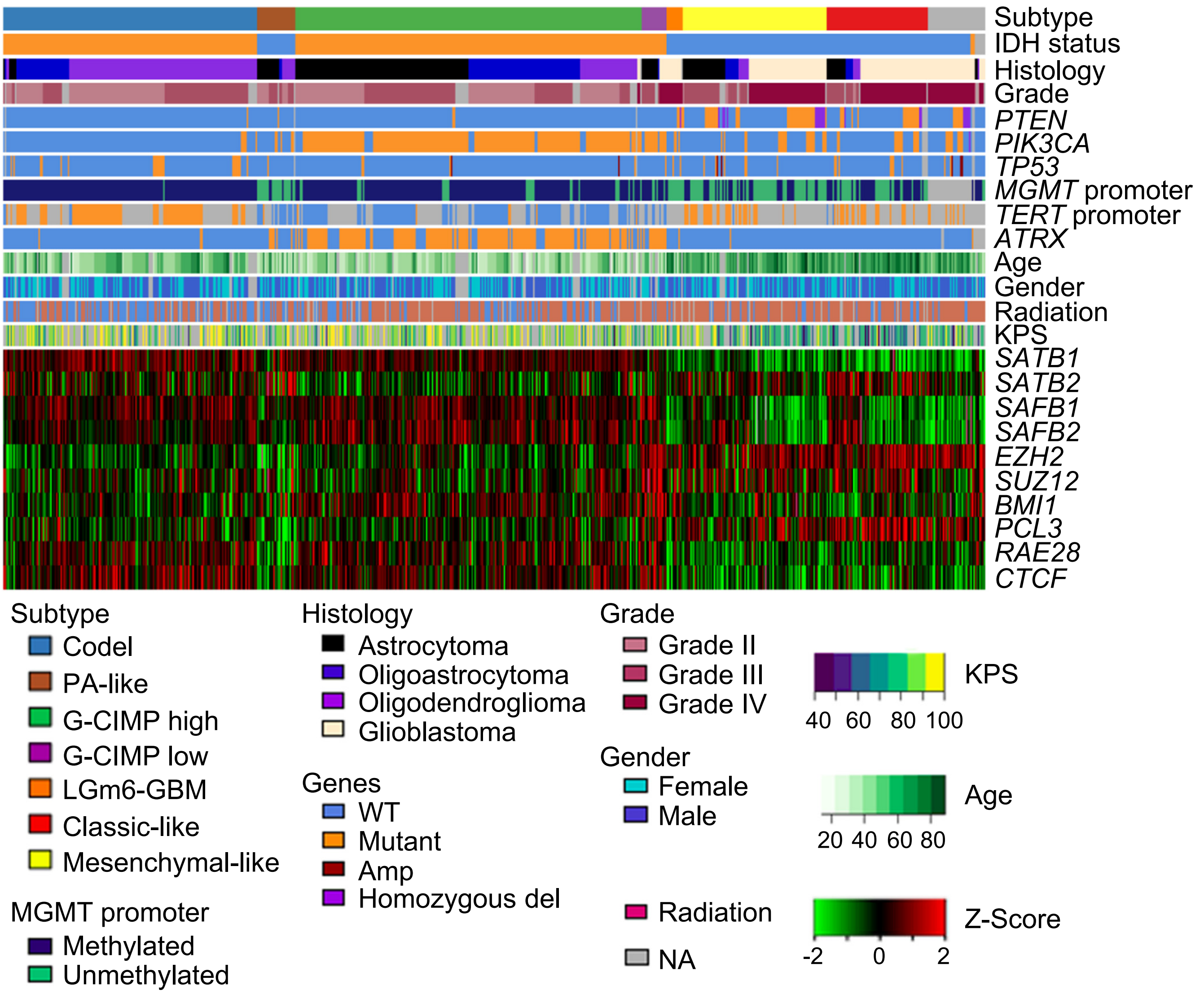
Subtype
IDH status
Histology
Grade
PTEN
PIK3CA
TP53
MGMT promoter
TERT promoter
ATRX
Age
Gender
Radiation
KPS
SATB1
SATB2
SAFB1
SAFB2
EZH2
SUZ12
BMI1
PCL3
RAE28
CTCF
Subtype
Codel
PA-like
G-CIMP high
G-CIMP low
LGm6-GBM
Classic-like
Mesenchymal-like
MGMT promoter
Methylated
Unmethylated
Histology
Astrocytoma
Oligoastrocytoma
Oligodendroglioma
Glioblastoma
Genes
WT
Mutant
Amp
Homozygous del
Grade
Grade II
Grade III
Grade IV
Gender
Female
Male
Radiation
NA
KPS
40 60 80 100
Age
20 40 60 80
Z-Score
-2 0 2

Appendix Figure S1. SATB2 is enriched in human GBMs.

Microarray and whole exome data as well as clinical phenotype were aggregated from TCGA GBM and low-grade glioma (LGG) databases to visualize the expression pattern of nuclear matrix-associated proteins (NMPs). Codel, codeletion of chromosomes 1p and 19q; PA-like, pilocytic astrocytoma–like; CIMP, glioma-CpG island methylator phenotype; LGm6-GBM, a subgroup of glioma enriched for histologic lowgrade gliomas that also contains a subset of tumors with GBM-defining histologic criteria; KPS, Karnofsky performance status.

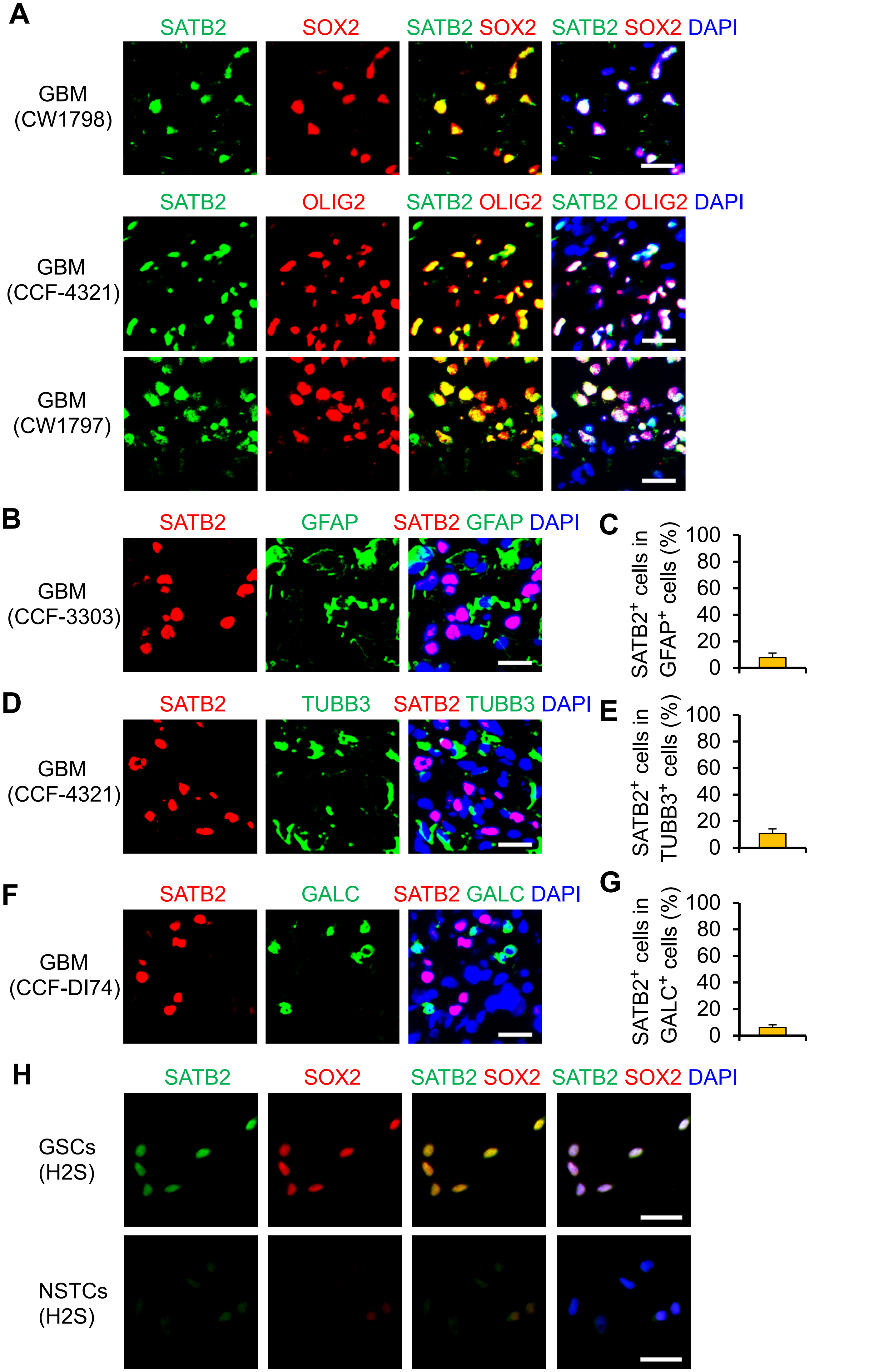
A
SATB2
SOX2
SATB2 SOX2
SATB2 SOX2 DAPI
GBM (CW1798)
SATB2
OLIG2
SATB2 OLIG2
SATB2 OLIG2 DAPI
GBM (CCF-4321)
GBM (CW1797)
B
SATB2
GFAP
SATB2 GFAP DAPI
GBM (CCF-3303)
C
SATB2+ cells in GFAP+ cells (%)
100
80
60
40
20
0
D
SATB2
TUBB3
SATB2 TUBB3 DAPI
GBM (CCF-4321)
E
SATB2+ cells in TUBB3+ cells (%)
100
80
60
40
20
0
F
SATB2
GALC
SATB2 GALC DAPI
GBM (CCF-DI74)
G
SATB2+ cells in GALC+ cells (%)
100
80
60
40
20
0
H
SATB2
SOX2
SATB2 SOX2
SATB2 SOX2 DAPI
GSCs (H2S)
NSTCs (H2S)

Appendix Figure S2. SATB2 is enriched in GSCs.

A. Immunofluorescence of SATB2 (green) and SOX2 or OLIG2 (red) on frozen tissue sections of human GBM surgical specimens. Scale bar, 25 μm.

B. Immunofluorescence of SATB2 (red) and the astrocyte marker GFAP (green) on frozen tissue sections of human GBM surgical specimens. Scale bar, 25 μm.

C. Quantification of the fraction of $SATB2^+$ cells in $GFAP^+$ cells in human GBMs. n=3 different GBMs.

D. Immunofluorescence of SATB2 (red) and the neuron marker TUBB3 (green) on frozen tissue sections of human GBM surgical specimens. Scale bar, 25 μm.

E. Quantification of the fraction of $SATB2^+$ cells in $TUBB3^+$ cells in human GBMs. n=3 different GBMs.

F. Immunofluorescence of SATB2 (red) and the oligodendrocyte marker GALC (green) on frozen tissue sections of human GBM surgical specimens. Scale bar, 25 μm.

G. Quantification of the fraction of $SATB2^+$ cells in $GALC^+$ cells in human GBMs. n=3 different GBMs.

H. Immunofluorescence of SATB2 (green) and SOX2 (red) in H2S GSCs and matched NSTCs. Scale bar, 50 μm.

Data information: Data are represented as mean ± SD.

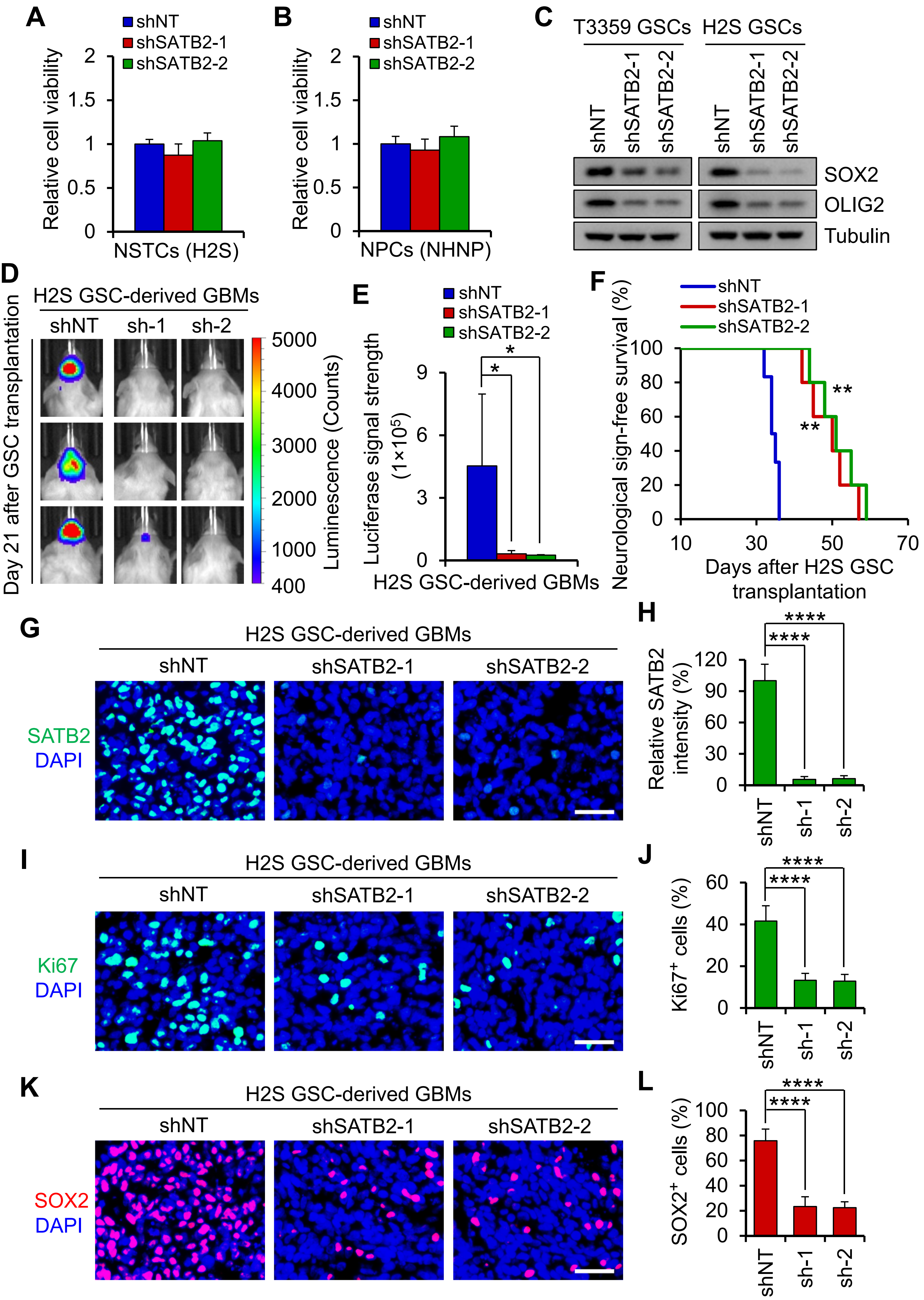
A
shNT
shSATB2-1
shSATB2-2
Relative cell viability
NSTCs (H2S)
B
NPCs (NHNP)
C
T3359 GSCs
H2S GSCs
SOX2
OLIG2
Tubulin
D
H2S GSC-derived GBMs
shNT
sh-1
sh-2
Day 21 after GSC transplantation
Luminescence (Counts)
E
Luciferase signal strength (1×10^5)
F
Neurological sign-free survival (%)
Days after H2S GSC transplantation
G
SATB2
DAPI
H
Relative SATB2 intensity (%)
I
Ki67
J
Ki67+ cells (%)
K
SOX2
L
SOX2+ cells (%)

Appendix Figure S3. Disrupting SATB2 suppressed GSC proliferation and GBM growth.

A. Cell viability of H2S NSTCs expressing shNT or shSATB2 at day 3 after cell seeding (n=5).

B. Cell viability of NHNP NPCs expressing shNT or shSATB2 at day 3 after cell seeding (n=5).

C. Immunoblot analysis of SOX2 and OLIG2 expression in GSCs transduced with shNT or shSATB2.

D. Bioluminescent images of the GBM xenografts derived from the luciferase-labeled H2S GSCs expressing shNT or shSATB2. Representative inmages on day 21 after transplantation are shown (n=5 mice per group).

E. Quantification of the bioluminescence of xenografts derived from the luciferase-labeled H2S GSCs expressing shNT or shSATB2 at day 21 posttransplantation (n=5 mice per group).

F. Kaplan-Meier survival curves of mice intracranially implanted with H2S GSCs expressing shNT or shSATB2 (shNT: n=6 mice; shSATB2-1 or shSATB2-2: n=5 mice). Median survival: shNT, 34.5 days; shSATB2-1, 50 days; shSATB2-2, 51 Days.

G. Immunofluorescence of SATB2 (Green) in GBM xenografts derived from H2S GSCs expressing shNT or shSATB2 (n=5 tumors per group). Scale bar: 40 μm.

H. Quantification of SATB2 intensity in xenografts derived from H2S GSCs expressing shNT or shSATB2 (n=5 tumors per group).

I. Immunofluorescence of Ki67 (Green) in tumor xenografts derived from H2S GSCs expressing shNT or shSATB2 (n=5 tumors per group). Scale bar: 40 μm.

J. Quantification of Ki67 positive cells in xenografts derived from H2S GSCs expressing shNT or shSATB2 (n=5 tumors per group).

K. Immunofluorescence of SOX2 (Red) in xenografts derived from H2S GSCs expressing shNT or shSATB2 (n=5 tumors per group). Scale bar: 40 μm.

L. Quantification of SOX2 positive cells in xenografts derived from H2S GSCs expressing shNT or shSATB2 (n=5 tumors per group).

Data information: Data are shown as mean ± SD. *$p < 0.5$, **$p < 0.01$, ****$p < 0.0001$ compared with shNT, one way ANOVA analysis followed by Tukey's test (A, B, H, J and L), Kruskal–Wallis test followed by Dunn's test (E), or log-rank test (F). Exact *p* values are reported in Appendix Table S3.

Appendix Fig S4

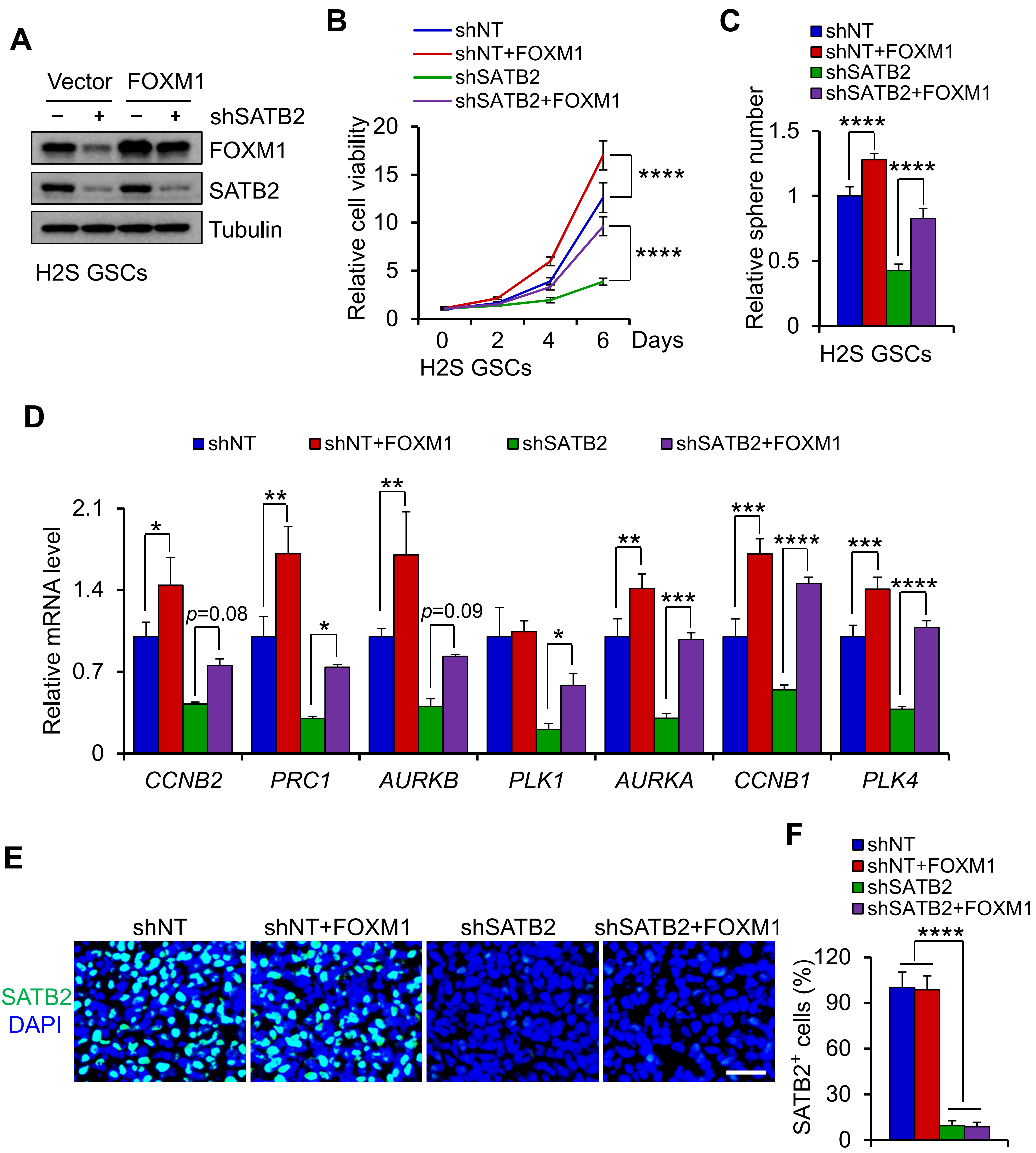

Appendix Figure S4. Ectopic expression of FOXM1 rescued the decreased GSC proliferation caused by SATB2 disruption.

A. Immunoblot analysis of FOXM1 and SATB2 expression in H2S GSCs transduced with FOXM1 or vector control in combination with shNT or shSATB2.

B. Cell viability assays of H2S GSCs transduced with FOXM1 or vector control in combination with shNT or shSATB2 (n=5).

C. Tumorsphere number of H2S GSCs transduced with FOXM1 or vector control in combination with shNT or shSATB2 (n=5).

D. qPCR analysis of FOXM1 downstream targets in H2S GSCs transduced with with FOXM1 or vector control in combination with shNT or shSATB2 (n=3). Ectopic expression of FOXM1 in GSCs expressing shSATB2 restored the expression of proliferative-related genes.

E. Immunofluorescence of SATB2 (Green) in xenografts derived from T3359 GSCs transduced with FOXM1 or vector control in combination with shNT or shSATB2 (n=5 tumors per group). Scale bar: 40 μm.

F. Quantification of SATB2 intensity in xenografts derived from T3359 GSCs transduced with FOXM1 or vector control in combination with shNT or shSATB2 (n=5 tumors per group).

Data information: Data are shown as mean ± SD. *$p<0.5$, **$p<0.01$, ***$p<0.001$, ****$p<0.0001$, two way ANOVA analysis followed by Tukey's test (B) or one way ANOVA analysis followed by Tukey's test (C, D and F). Exact *p* values are reported in Appendix Table S3.

Appendix Fig S5

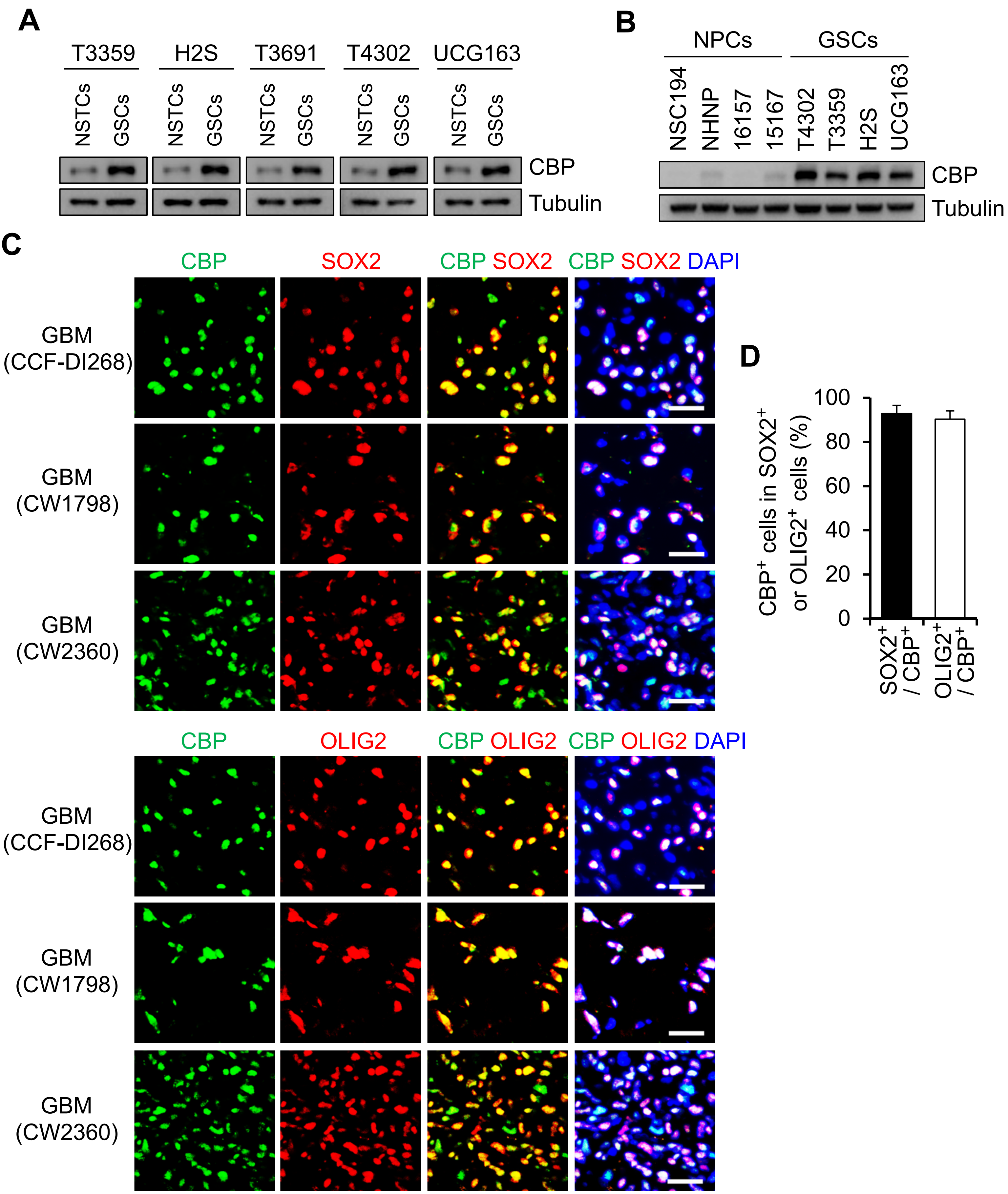

Appendix Figure S5. CBP is preferentially expressed by GSCs.

A. Immunoblot analysis of CBP expression in cell lysates of GSCs and matched NSTCs.

B. Immunoblot analysis of CBP expression in cell lysates of GSCs and NPCs.

C. Immunofluorescence of CBP (green) and the GSC marker SOX2 or OLIG2 (red) in human GBM specimens. CBP is preferentially expressed by GSCs in human GBMs. Scale bar, 25 μm.

D. Quantification of (C) showing the fraction of CBP^+ cells in $SOX2^+$ or $OLIG2^+$ cells in human GBMs. More than 90% $SOX2^+$ or $OLIG2^+$ cells showed CBP staining. n=3 GBMs.

Data information: Data are shown as mean ± SD.

Appendix Fig S6

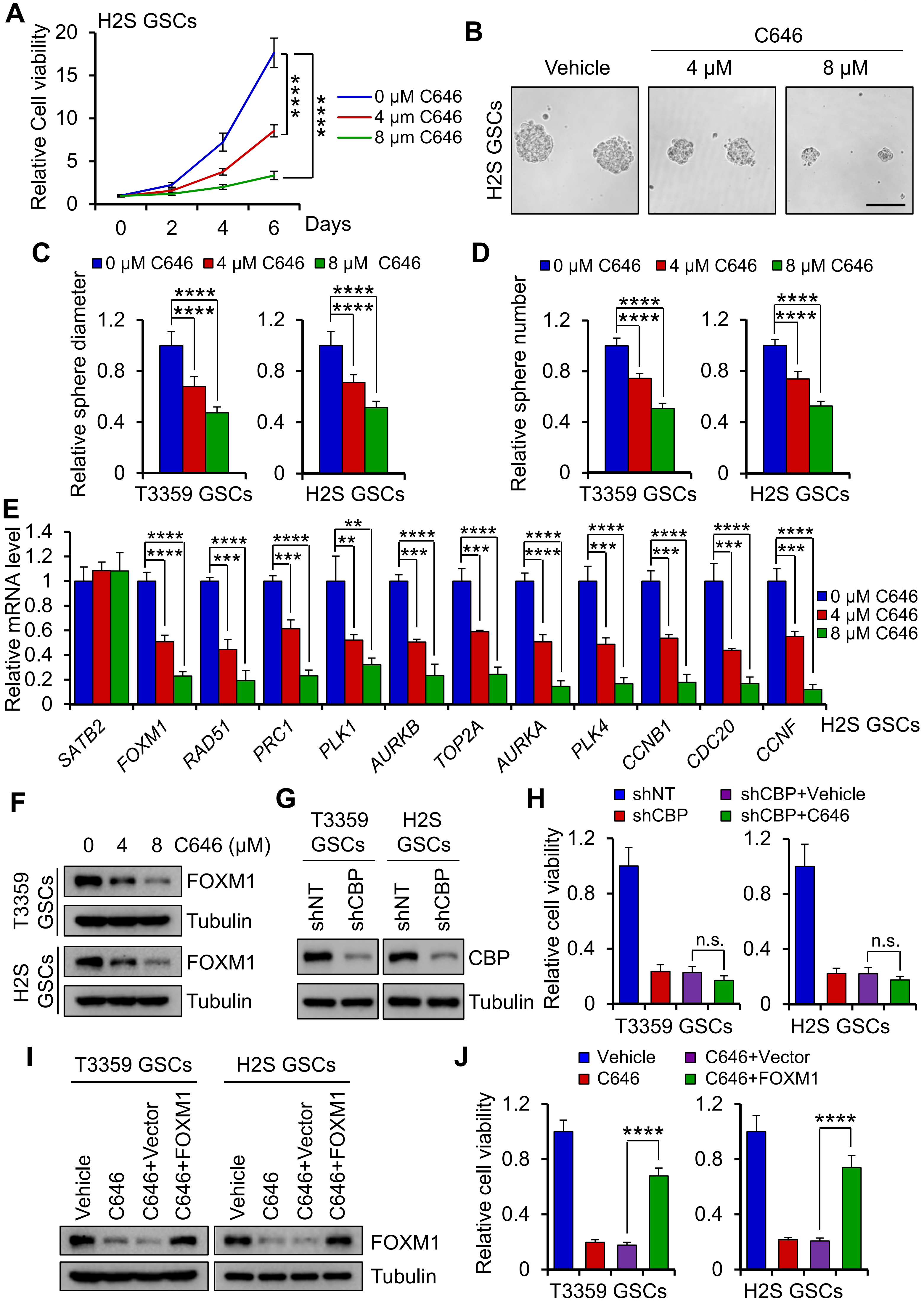

Appendix Figure S6. CBP inhibition by C646 impaired GSC proliferation.

A. Cell viability of H2S GSCs treated with indicated doses of C646 or the vehicle control (n=5).

B. Tumorsphere images of H2S GSCs treated with indicated doses of C646 or the vehicle control for 6 days. Scale bar: 100 μm.

C, D. Quantification of the diameter (C) or number (D) of tumorspheres from GSCs treated with indicated doses of C646 or the vehicle control for 6 days (C: n=9; D: n=4).

E. qPCR analysis of *SATB2*, *FOXM1* and FOXM1 downstream targets in H2S GSCs treated with indicated doses of C646 or the vehicle control for 24 hours (n=3).

F. Immunoblot analysis of FOXM1 expression in GSCs treated with indicated dose of C646 for 24 hours.

G. Immunoblot analysis of CBP expression in GSCs transduced with shCBP or shNT control for 48 hours.

H. Cell viability assay of GSCs transduced with shCBP or shNT for 48 hours and then treated with 8 μM C646 or the vehicle control for 6 days (n=5).

I. Immunoblot analysis of FOXM1 expression in GSCs transduced with FOXM1 or vector control and treated with 8 μM of C646 or the vehicle control for 48 hours.

J. Cell viability assay of GSCs transduced with FOXM1 or vector control and treated with 8 μM of C646 or the vehicle control for 6 days (n=5).

Data information: Data are represented as mean ± SD. **$p < 0.01$, ***$p < 0.001$, ****$p < 0.0001$, two way ANOVA analysis followed by Tukey's test (A) or one way ANOVA analysis followed by Tukey's test (C, D, E, H and J). Exact *p* values are reported in Appendix Table S3.

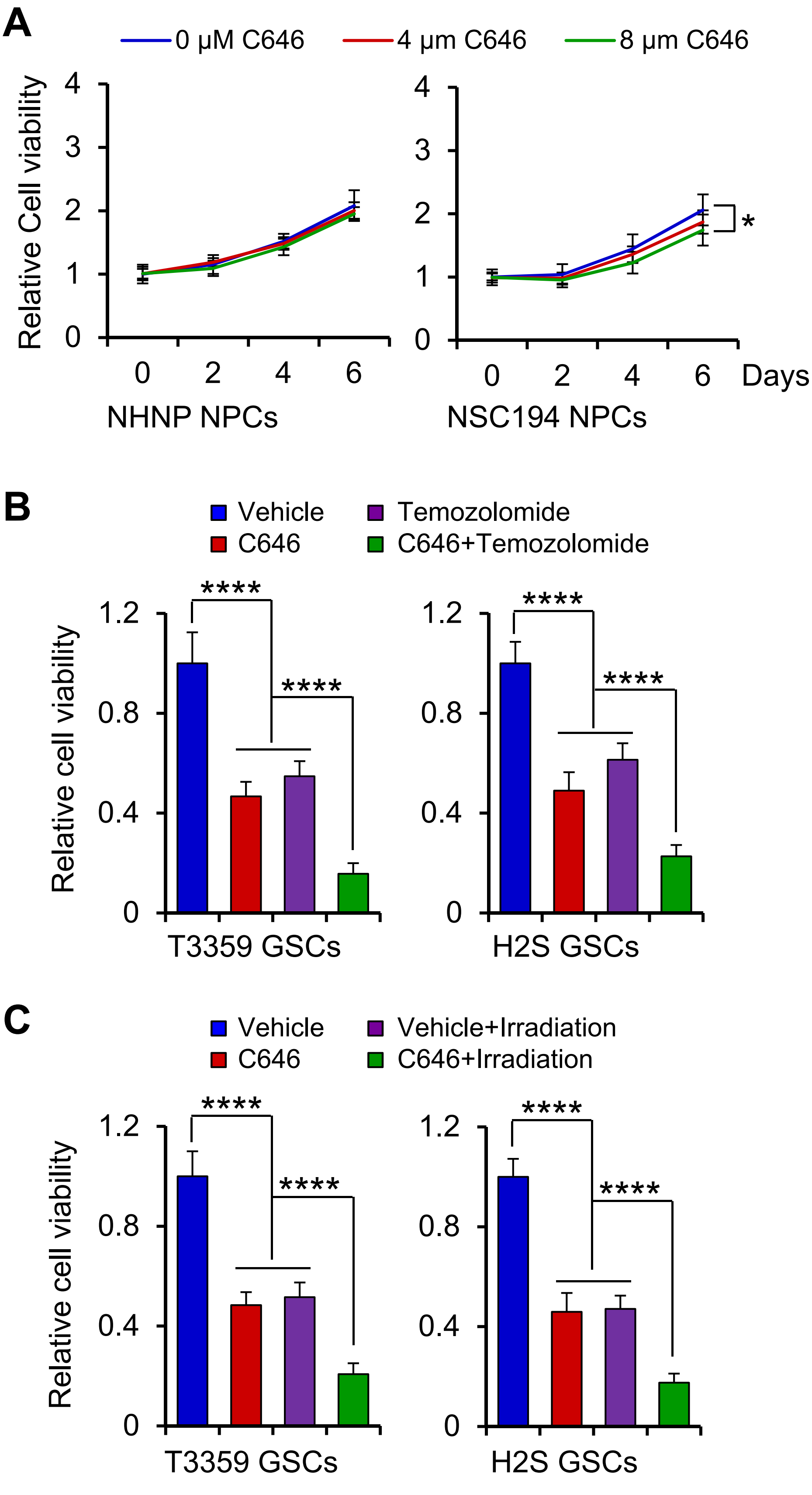
A
0 μM C646
4 μm C646
8 μm C646
Relative Cell viability
NHNP NPCs
NSC194 NPCs
Days
*
B
Vehicle
Temozolomide
C646
C646+Temozolomide
Relative cell viability
****
T3359 GSCs
H2S GSCs
C
Vehicle
Vehicle+Irradiation
C646
C646+Irradiation
Relative cell viability
****
T3359 GSCs
H2S GSCs

Appendix Figure S7. The CBP inhibitor C646 had little effect on NPC proliferation but had synergistic effect with Temozolomide or irradiation to inhibit GSC proliferation.

A. Cell viability of NPCs treated with indicated doses of C646 or the vehicle control (n=5).

B. Cell viability assay of GSCs treated with C646 (4 μM) or Temozolomide (40 μM) or in combination for 6 days (n=5).

C. Cell viability assay of GSCs treated with C646 (4 μM) or irradiation (1 Gy) or in combination for 6 days (n=5).

Data information: Data are represented as mean ± SD. $^{*}p < 0.05$, $^{****}p < 0.0001$, two way ANOVA analysis followed by Tukey's test (A) or one way ANOVA analysis followed by Tukey's test (B and C). Exact *p* values are reported in Appendix Table S3.

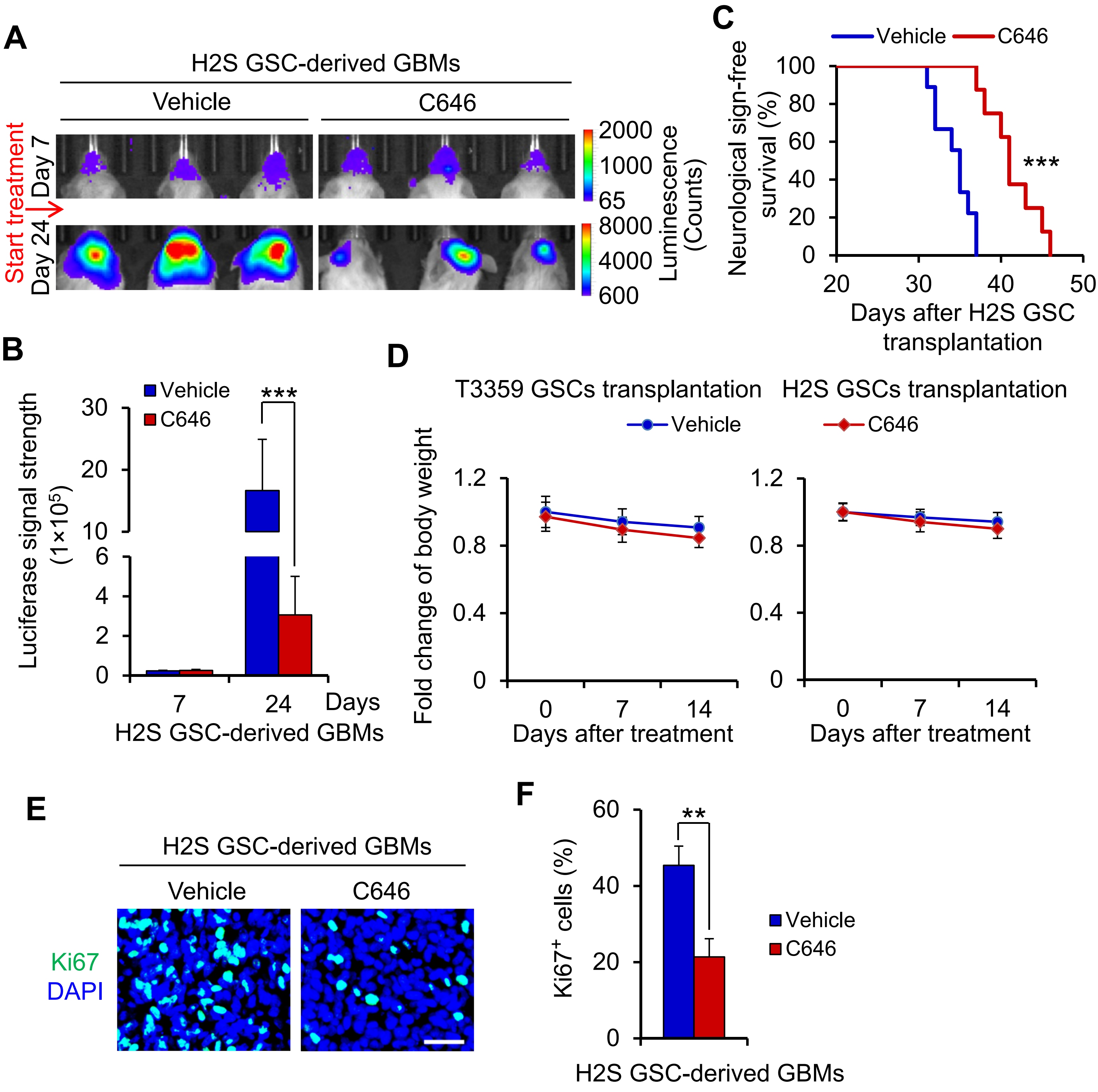
A
H2S GSC-derived GBMs
Vehicle
C646
Start treatment
Day 7
Day 24
2000
1000
65
8000
4000
600
Luminescence (Counts)
C
Vehicle
C646
Neurological sign-free survival (%)
100
80
60
40
20
0
***
20
30
40
50
Days after H2S GSC transplantation
B
Vehicle
C646
***
Luciferase signal strength (1×10^5)
30
20
10
6
4
2
0
7
24
Days
H2S GSC-derived GBMs
D
T3359 GSCs transplantation
H2S GSCs transplantation
Vehicle
C646
Fold change of body weight
1.2
0.8
0.4
0
0
7
14
Days after treatment
E
H2S GSC-derived GBMs
Vehicle
C646
Ki67
DAPI
F
60
40
20
0
**
Ki67+ cells (%)
Vehicle
C646
H2S GSC-derived GBMs

Appendix Figure S8. Targeting CBP by C646 impaired GSC-driven tumor growth.

A. Bioluminescent imaging of tumor growth in mice bearing xenografts derived from the luciferase-labeled H2S GSCs treated with C646 or the vehicle control at indicated days after GSC transplantation (n=5 mice per group).

B. Quantification of tumor growth from (A) (n=5 mice per group).

C. Kaplan-Meier survival curves of mice bearing H2S GSC-derived xenografts treated with C646 or the vehicle control (Vehicle control: n=9 mice; C646 treatment: n=8 mice). Median survival: Vehicle control, 35 days; C646, 41 days.

D. Relative body weight of mice bearing GSC-derived tumor xenografts treated with C646 or the vehicle control at indicated days (Vehicle control: n=9 mice; C646: n=8 mice).

E. Immunofluorescence of Ki67 (Green) in H2S GSC-derived xenografts from mice treated with with C646 or the vehicle control (n=6 tumors per group). Scale bar: 40 μm.

F. Quantification of Ki67 positive cells in H2S GSC-derived xenografts from mice treated with with C646 or the vehicle control (n=6 tumors per group).

Data information: Data are shown as mean ± SD. **$p < 0.01$, ***$p < 0.001$, two way ANOVA analysis followed by Sidak's test (B and D), log-rank test (C), or Mann-Whitney test (F). Exact *p* values are reported in Appendix Table S3.

Appendix Table S1. *In vivo* limiting dilution assay for tumor formation of GSCs expressing shNT or shSATB2. Tumor incidence, median survival time of mice and stem cell frequency after intracranial transplantation of 5000, 1000, 500 or 100 GSCs (T3359) expressing shNT, shSATB2-1, or shSATB2-2. $^{**}p < 0.01$ with Log-rank analysis of survival curves for the same number of GSCs expressing shSATB2 relative to shNT control. $^{***}p < 0.001$, $^{****}p < 0.0001$ with ELDA analysis of stem cell frequencies between shNT and shSATB2 groups. Exact *p* values are reported in Appendix Table S3.

shRNA	GSC number	5000	1000	500	100	Stem cell frequency
shNT	Incidence<br>Median survival	5/5<br>29	5/5<br>36	5/5<br>41	5/5<br>57	1/1
shSATB2-1	Incidence<br>Median survival	5/5<br>49**	5/5<br>62**	3/5<br>73**	0/5<br>---**	1/475****
shSATB2-2	Incidence<br>Median survival	5/5<br>47**	5/5<br>58**	3/5<br>67**	1/5<br>---**	1/400***

Appendix Table S2. List of qPCR primers.

Gene	Forward/Reverse primer
18S rRNA	5'-AACCCGTTGAACCCCATT-3'
	5'-CCATCCAATCGGTAGTAGCG-3'
SATB2	5'-GGAGAACGACAGCGAGGAA-3'
	5'-CCGATGTATTGCTTTGCCTAGT-3'
FOXM1	5'-ATACGTGGATTGAGGACCACT-3'
	5'-TCCAATGTCAAGTAGCGGTTG-3'
CCNB2	5'-TGCTCTGCAAAATCGAGGACA-3'
	5'- GCCAATCCACTAGGATGGCA-3'
RAD51	5'-CAACCCATTTCACGGTTAGAGC-3'
	5'-TTCTTTGGCGCATAGGCAACA-3'
FANCI	5'-CCACCTTTGGTCTATCAGCTTC-3'
	5'-CAACATCCAATAGCTCGTCACC-3'
PRC1	5'-AAGTGGAATTGATGCGAAAACAG-3'
	5'-CCTCACGCCTAGAAGCCTTTG-3'
PLK1	5'-AAAGAGATCCCGGAGGTCCTA-3'
	5'-GGCTGCGGTGAATGGATATTTC-3'
AURKB	5'-CAGAAGAGCTGCACATTTGACG-3'
	5'-CCTTGAGCCCTAAGAGCAGATTT-3'
TOP2A	5'-TTAATGCTGCGGACAACAAACA-3'
	5'-CGACCACCTGTCACTTTCTTTT-3'
UBE2C	5′-CGTGTTCTCCGAGTTCCTGT-3′
	5′-GCTCCTGCTGTAGCCTTTTG-3′
AURKA	5’-GAGGTCCAAAACGTGTTCTCG-3’
	5’-ACAGGATGAGGTACACTGGTTG-3’
PLK4	5′-AAGCTCGACACTTCATGCACC-3′
	5′-GCATTTTCAGTTGAGTTGCCAG-3′
KIF20A	5′-TTGAGGGTTAGGCCCTTGTTA-3′
	5′-GTCCTTGGGTGCTTGTAGAAC-3′
CCNB1	5′-AATAAGGCGAAGATCAACATGGC-3′
	5′-TTTGTTACCAATGTCCCCAAGAG-3′
CDC20	5'- GACCACTCCTAGCAAACCTGG-3'
	5'-GGGCGTCTGGCTGTTTTCA-3'
CCNF	5′-GGAAAGCGACAGGAGGACAG-3′
	5′-TGGCAGACGATCTCACTGGAA-3′
p21	5′-TCCAGCGACCTTCCTCATCCAC-3′
	5′-TCCATAGCCTCTACTGCCACCATC-3′
p27	5′-TAATTGGGGCTCCGGCTAACT-3′
	5′-TGCAGGTCGCTTCCTTATTCC-3′

Appendix Table S3. Summary of statistical tests and *p* values.

Figure number	Test used	Comparison	*p* value
Fig 1C	Mann-Whitney test	H2S NSTCs vs. GSCs	0.0079
		T3359 NSTCs vs. GSCs	0.0079
		T3691 NSTCs vs. GSCs	0.0079
		T4302 NSTCs vs. GSCs	0.0079
		UCG163 NSTCs vs. GSCs	0.0079
Fig 1F	One way ANOVA analysis followed by Tukey's test	NSC194 vs. T3359	<0.0001
		NSC194 vs. H2S	<0.0001
		NSC194 vs. UCG163	<0.0001
		NHNP vs. T3359	<0.0001
		NHNP vs. H2S	<0.0001
		NHNP vs. UCG163	<0.0001
		16157 vs. T3359	0.0001
		16157 vs. H2S	<0.0001
		16157 vs. UCG163	<0.0001
Fig 2B	Two way ANOVA analysis followed by Tukey's test		<0.0001
Fig 2D	One way ANOVA analysis followed by Tukey's test		<0.0001
Fig 2F	One way ANOVA analysis followed by Tukey's test		<0.0001
Fig 2G	One way ANOVA analysis followed by Tukey's test		<0.0001
Fig 2H	ELDA analysis	T3359 shNT vs. sh-1	1.29E-20
		T3359 shNT vs. sh-2	7.38E-25
		H2S shNT vs. sh-1	7.74E-14
		H2S shNT vs. sh-2	1.47E-19
Fig 3B	Kruskal–Wallis test followed by Dunn's test	shNT vs. sh-1	0.0175
		shNT vs. sh-2	0.0327
Fig 3C	Log-rank test	shNT vs. sh-1	0.001
		shNT vs. sh-2	0.001
Fig 3E	One way ANOVA analysis followed by Tukey's test		<0.0001
Fig 3G	One way ANOVA analysis followed by Tukey's test		<0.0001
Fig 3I	One way ANOVA analysis followed by Tukey's test		<0.0001
Fig 4C	One way ANOVA analysis followed by Tukey's test		<0.0001
Fig 4G	One way ANOVA analysis followed by Tukey's test	CCNB2 shNT vs. sh-1	0.0011
		shNT vs. sh-2	0.0008
		RAD51 shNT vs. sh-1	<0.0001
		shNT vs. sh-2	<0.0001
		FANCI shNT vs. sh-1	<0.0001
		shNT vs. sh-2	<0.0001
		PRC1 shNT vs. sh-1	<0.0001
		shNT vs. sh-2	<0.0001

		PLK1 shNT vs. sh-1	0.0023
		shNT vs. sh-2	0.0006
		AURKB shNT vs. sh-1	0.0008
		shNT vs. sh-2	0.0005
		TOP2A shNT vs. sh-1	<0.0001
		shNT vs. sh-2	<0.0001
		UBE2C shNT vs. sh-1	0.0009
		shNT vs. sh-2	0.0047
		AURKA shNT vs. sh-1	<0.0001
		shNT vs. sh-2	<0.0001
		PLK4 shNT vs. sh-1	0.0003
		shNT vs. sh-2	0.0002
		KLF20A shNT vs. sh-1	<0.0001
		shNT vs. sh-2	<0.0001
		CCNB1 shNT vs. sh-1	0.0002
		shNT vs. sh-2	0.0003
		CDC20 shNT vs. sh-1	0.0001
		shNT vs. sh-2	0.0002
		CCNF shNT vs. sh-1	<0.0001
		shNT vs. sh-2	<0.0001
		p21 shNT vs. sh-1	0.0012
		shNT vs. sh-2	0.0013
		p27 shNT vs. sh-1	0.0403
		shNT vs. sh-2	0.0185
Fig 5B	Two way ANOVA analysis followed by Tukey's test		<0.0001
Fig 5C	One way ANOVA analysis followed by Tukey's test		<0.0001
Fig 5E	One way ANOVA analysis followed by Tukey's test	shNT vs. shNT+FOXM1	0.0014
		shSATB2 vs. shSATB2+FOXM1	<0.0001
Fig 5F	Log-rank test	1 vs. 2	0.0034
		1 vs. 3	0.0003
		3 vs. 4	0.0003
Fig 5H	One way ANOVA analysis followed by Tukey's test	shNT vs. shNT+FOXM1	0.0819
		shSATB2 vs. shSATB2+FOXM1	0.0033
Fig 5J	One way ANOVA analysis followed by Tukey's test	shNT vs. shNT+FOXM1	0.0742
		shSATB2 vs. shSATB2+FOXM1	<0.0001
Fig 6C	One way ANOVA analysis followed by Tukey's test	T3359 shNT vs. sh-1	0.0003
		shNT vs. sh-2	0.0005
		H2S shNT vs. sh-1	0.0134
		shNT vs. sh-2	0.0123
Fig 6E	One way ANOVA analysis followed by Tukey's test		<0.0001
Fig 6F	One way ANOVA analysis followed by Tukey's test	AcH3K18 shNT vs. sh-1	0.0021
		shNT vs. sh-2	0.0026
		AcH3K27 shNT vs. sh-1	0.0017
		shNT vs. sh-2	0.0056
		AcH4 shNT vs. sh-1	0.0011
		shNT vs. sh-2	0.0024

Fig 6G	One way ANOVA analysis followed by Tukey's test	Control vs. shSATB2	<0.0001
		Control vs. shCBP	<0.0001
		shSATB2 vs. shSATB2+shCBP	0.0006
		shCBP vs. shSATB2+shCBP	0.0002
Fig 7A	Two way ANOVA analysis followed by Tukey's test		<0.0001
Fig 7C	One way ANOVA analysis followed by Tukey's test	FOXM1 shNT vs. sh-1	0.0012
		shNT vs. sh-2	<0.0001
		RAD51 shNT vs. sh-1	0.0009
		shNT vs. sh-2	<0.0001
		PRC1 shNT vs. sh-1	0.0004
		shNT vs. sh-2	<0.0001
		PLK1 shNT vs. sh-1	0.0344
		shNT vs. sh-2	0.0072
		AURKB shNT vs. sh-1	<0.0001
		shNT vs. sh-2	<0.0001
		TOP2A shNT vs. sh-1	0.0005
		shNT vs. sh-2	0.0001
		AURKA shNT vs. sh-1	0.002
		shNT vs. sh-2	<0.0001
		PLK4 shNT vs. sh-1	0.0039
		shNT vs. sh-2	0.0001
		CCNB1 shNT vs. sh-1	0.0206
		shNT vs. sh-2	0.0047
		CDC20 shNT vs. sh-1	<0.0001
		shNT vs. sh-2	<0.0001
		CCNF shNT vs. sh-1	0.0026
		shNT vs. sh-2	<0.0001
Fig 7F	Two way ANOVA analysis followed by Sidak's test		<0.0001
Fig 7G	Log-rank test		<0.0001
Fig 7I	Mann-Whitney test		0.0022
Appendix Fig S3E	Kruskal–Wallis test followed by Dunn's test	shNT vs. sh-1	0.04
		shNT vs. sh-2	0.014
Appendix Fig S3F	Log-rank test	shNT vs. sh-1	0.0018
		shNT vs. sh-2	0.0018
Appendix Fig S3H	One way ANOVA analysis followed by Tukey's test		<0.0001
Appendix Fig S3J	One way ANOVA analysis followed by Tukey's test		<0.0001
Appendix Fig S3L	One way ANOVA analysis followed by Tukey's test		<0.0001
Appendix Fig S4B	Two way ANOVA analysis followed by Tukey's test		<0.0001
Appendix Fig S4D	One way ANOVA analysis followed by Tukey's test	CCNB2 shNT vs. shNT+FOXM1	0.0189
		CCNB2 shSATB2 vs. shSATB2+FOXM1	0.0752
		PRC1 shNT vs. shNT+FOXM1	0.0015
		PRC1 shSATB2 vs. shSATB2+FOXM1	0.0249

		AURKB shNT vs. shNT+FOXM1	0.0084
		AURKB shSATB2 vs. shSATB2+FOXM1	0.0942
		PLK1 shSATB2 vs. shSATB2+FOXM1	0.0497
		AURKA shNT vs. shNT+FOXM1	0.0062
		AURKA shSATB2 vs. shSATB2+FOXM1	0.0002
		CCNB1 shNT vs. shNT+FOXM1	0.0002
		CCNB1 shSATB2 vs. shSATB2+FOXM1	<0.0001
		PLK4 shNT vs. shNT+FOXM1	0.0009
		PLK4 shSATB2 vs. shSATB2+FOXM1	<0.0001
Appendix Fig S4F	One way ANOVA analysis followed by Tukey's test		<0.0001
Appendix Fig S6A	Two way ANOVA analysis followed by Tukey's test		<0.0001
Appendix Fig S6C	One way ANOVA analysis followed by Tukey's test		<0.0001
Appendix Fig S6D	One way ANOVA analysis followed by Tukey's test		<0.0001
Appendix Fig S6E	One way ANOVA analysis followed by Tukey's test	FOXM1 shNT vs. sh-1	<0.0001
		shNT vs. sh-2	<0.0001
		RAD51 shNT vs. sh-1	0.0002
		shNT vs. sh-2	<0.0001
		PRC1 shNT vs. sh-1	0.0003
		shNT vs. sh-2	<0.0001
		PLK1 shNT vs. sh-1	0.0076
		shNT vs. sh-2	0.0013
		AURKB shNT vs. sh-1	0.0002
		shNT vs. sh-2	<0.0001
		TOP2A shNT vs. sh-1	0.0007
		shNT vs. sh-2	<0.0001
		AURKA shNT vs. sh-1	<0.0001
		shNT vs. sh-2	<0.0001
		PLK4 shNT vs. sh-1	0.0006
		shNT vs. sh-2	<0.0001
		CCNB1 shNT vs. sh-1	0.0003
		shNT vs. sh-2	<0.0001
		CDC20 shNT vs. sh-1	0.0006
		shNT vs. sh-2	<0.0001
		CCNF shNT vs. sh-1	0.0004
		shNT vs. sh-2	<0.0001
Appendix Fig S6J	One way ANOVA analysis followed by Tukey's test		<0.0001
Appendix Fig S7A	Two way ANOVA analysis followed by Tukey's test		0.0194
Appendix Fig S7B	One way ANOVA analysis followed by Tukey's test		<0.0001
Appendix Fig S7C	One way ANOVA analysis followed by Tukey's test		<0.0001
Appendix Fig S8B	Two way ANOVA analysis followed by Sidak's test		0.0002

Appendix Fig S8C	Log-rank test		0.0001
Appendix Fig S8F	Mann-Whitney test		0.0022
Appendix Table S1	ELDA analysis (Stem cell frequency)	shNT vs. sh-1	8.41E-05
		shNT vs. sh-2	2.04E-04
	Log-rank test (Survival curve)	5000 shNT vs. sh-1	0.0018
		shNT vs. sh-2	0.0018
		1000 shNT vs. sh-1	0.0018
		shNT vs. sh-2	0.0018
		500 shNT vs. sh-1	0.0018
		shNT vs. sh-2	0.0018
		100 shNT vs. sh-1	0.0018
		shNT vs. sh-2	0.0064
